# What is the rhythm?

**DOI:** 10.1007/s12471-018-1155-8

**Published:** 2018-09-03

**Authors:** A. W. G. J. Oomen, R. W. Sy

**Affiliations:** 1 Department of Cardiology, Royal Prince Alfred Hospital, Camperdown, NSW Australia; 2 Sydney Medical School, University of Sydney, Sydney, NSW Australia

## Answer

**Fig. 1:**
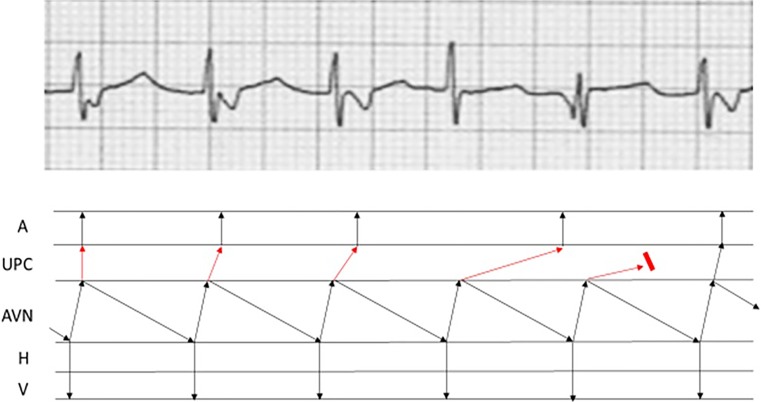
Ladder diagram showing presumed mechanism of tachycardia: typical AV nodal re-entrant tachycardia with Wenckebach phenomenon in upper common final pathway

The ECG (Fig. 1 in the question) shows a regular narrow complex tachycardia at a rate of approximately 150 beats/min starting after the second QRS complex. There are more QRS complexes than P waves. The P waves have a superior axis and there are also variable P‑P intervals.

Atrial tachycardia is excluded because there are more QRS complexes than P waves. The absence of a 1:1 atrioventricular (AV) relationship also excludes orthodromic AV re-entrant tachycardia. The ECG can only be explained by rare types of supraventricular tachycardia which can manifest with either complete dissociation or intermittent conduction block to the atria. These include junctional tachycardia, AV nodal re-entrant tachycardia and nodoventricular or nodofascicular re-entrant tachycardia. Of these, AV nodal re-entrant tachycardia would be the most likely diagnosis on the basis of probability alone. Junctional tachycardia occurs infrequently in adults, except in the context of cardiac surgery or digoxin toxicity. Nodoventricular or nodofascicular re-entrant tachycardia are extremely rare. An electrophysiology study is required to differentiate these rarer entities from AV nodal re-entrant tachycardia. For example, initiation with a critical atrial-His delay would not be expected in junctional tachycardia, and His-synchronous ventricular ectopy would perturb nodoventricular or nodofascicular re-entrant tachycardia.

This patient was referred for an electrophysiology study and she was found to have AV nodal re-entrant tachycardia for which she underwent a slow pathway ablation. The patient has been arrhythmia-free following ablation.

The mechanism of the tachycardia was most likely AV nodal re-entrant tachycardia with Wenckebach phenomenon in the upper common final pathway. This is depicted in Fig. 1.

